# Limited indirect effects of an infant pneumococcal vaccination program in an aging population

**DOI:** 10.1371/journal.pone.0220453

**Published:** 2019-08-01

**Authors:** Mark van der Linden, Matthias Imöhl, Stephanie Perniciaro

**Affiliations:** National Reference Center for Streptococci, Department of Medical Microbiology, University Hospital (RWTH), Aachen, Germany; Universidade de Lisboa Faculdade de Medicina, PORTUGAL

## Abstract

**Background:**

A general recommendation for adult pneumococcal vaccination with 23-valent polysaccharide vaccine (PPV23) for adults 60 and older has been in place in Germany since 1998, but uptake has been low. Just over a decade after the implementation of an infant pneumococcal conjugate vaccine recommendation, we examined indirect protection effects on adult invasive pneumococcal disease (IPD) in Germany.

**Methods and findings:**

Reported IPD cases decreased in children under two years of age from 11.09 per 100,000 in 2003–2006 to 5.94 per 100,000 in 2017/18, while in adult age groups, reported IPD cases rose across the board, most dramatically in adults 60 years of age and over, from 1.64 to 10.08 cases per 100,000. PCV13-type IPD represents 31% of all cases in this age group, the lion’s share of which is due to the rapid increase of serotype 3 IPD, which, by itself, has reached 2.11 reported cases per 100,000 and makes up 21% of all IPD cases in this age group. The two vaccine formulations currently in development (PCV15 and PCV20) would increase current (PCV13) coverage by 8.5% points and 28.0% points in children, while in adults coverage would increase by 10.4% points and 21.9% points, respectively.

**Conclusions:**

While original models predicted that indirect effects of childhood vaccination would suffice for adults, it seems that the herd protection effect has reached its limit, with vaccine serotypes 4, 19F, and 19A IPD persisting in adults after initial reductions, and serotype 3 IPD not showing any herd protection effect at all.

## Introduction

Even in the age of widespread pneumococcal vaccination, *Streptococcus pneumoniae* (pneumococcus) persists as a major cause of infectious disease worldwide [1]. Apart from causing invasive and non-invasive disease, pneumococci also colonize the nasopharynx. This asymptomatic carriage is most prevalent among young children (up to 50% among children under 2 years of age) but also present, although to a much lower extent (2–5%), among adults [2, 3]. Colonization is assumed to precede disease with transmission occurring among children, from children to adults, but also from one adult to another [4, 5]. The main burden of pneumococcal disease is found among children under 5 years of age, and among adults 50 years and older.

The first whole cell pneumococcal vaccines became available more than 100 years ago and were soon followed by vaccines containing capsular polysaccharides. Even though it was reasonably effective, pneumococcal polysaccharide vaccination was abandoned at the advent of antibiotics, in the 1940s, only to become important again, when antibiotic resistant pneumococci became more and more prevalent in the 1970s. A 23-valent polysaccharide vaccine (PPV23, serotypes 1, 2, 3, 4, 5, 6B, 7F, 8, 9N, 9V, 10A, 11A, 12F, 14, 15B, 17F, 18C, 19F, 19A, 20, 22F, 23F, and 33F) has been available since 1983, and is still recommended in many countries worldwide. As polysaccharide vaccines are not effective in children under the age of two, pneumococcal conjugate vaccines were developed, linking the polysaccharide to a protein carrier [6]. Conjugate vaccines are effective in young children, not only against invasive and non-invasive pneumococcal disease, but also, at least in some capacity, against carriage [7–9]. The first pneumococcal conjugate vaccine (PCV7) contained 7 of the most prevalent serotypes among invasive pneumococcal disease (4, 6B, 9V, 14, 18C, 19F and 23F) and was introduced 2000 (discontinued in 2017). Currently, two PCVs are available, PCV10 and PCV13 containing antigens for 10 (PCV7 + 1, 5, 7F) and 13 (PCV7 + 1, 5, 7F, 3, 6A, 19A) serotypes repectively.

The polysaccharide capsular is the main virulence factor of *S*. *pneumoniae* and currently over 94 different serotypes types have been described. Pneumococci with different serotypes differ in their capacities to colonize and to invade resulting in large differences in prevalence within pneumococcal disease [10, 11]. The serotypes included in the different vaccine formulations have been selected largely on basis of their prevalence among invasive disease.

Since pneumococcal conjugate vaccines have become available, many countries have introduced childhood vaccination programs. These have not only resulted in disease reduction among vaccinated children but also among non-vaccinated children, through a phenomenon called herd protection [12, 13]. Due to the fact that conjugate vaccines elicit mucosal immunity, vaccine serotype acquisition in the nasopharynx will become less likely among vaccinated children. Therefore, the transfer of these serotypes towards other (vaccinated and non-vaccinated) individuals in the community is interrupted. As children carrying pneumococci can also transfer their pneumococci to older individuals, the serotype distribution among pneumococcal disease in the elderly has also changed considerably, with vaccine types disappearing to a large extent. Except for in the US, the herd protection effect has not led to strong reductions of the disease burden among adults [14–16]. Long before the introduction of childhood conjugate vaccination, many countries had a vaccination recommendation for pneumococcal polysaccharide vaccine in place. However, these programs have had limited effects on pneumococcal disease among adults, also because vaccination rates remained very low [17].

The European Medicines Agency (EMA) licenced PCV13 for use in adults 50 years and older in 2011. However, only a few countries decided to include the vaccine into their adult recommendation, mostly in association with sequential PPV23 [18, 19]. Similarly, in Germany, the recommendation for PPV23 vaccination for all adults 60 years and older has remained [20]. Part of the reasoning of the recommending body (STIKO) was that the PCV13 serotypes were already targeted in childhood vaccination and would disappear among adults through herd protection, as was predicted by a health-economic model [21].

In Germany, pneumococcal conjugate vaccination for all children under the age of two has been recommended since June 2006, with a 3+1 vaccination schedule, changed to a 2+1 schedule in August 2015 [22]. Higher valency vaccines were introduced in April 2009 (PCV10) and December 2009 (PCV13), with the choice of vaccine lying with the parents and pediatricians, and costs fully reimbursed by health insurance. Uptake of PCV is 85% as measured in 2018 among children at school entry age [23]

A recommendation for adult vaccination with PPV23 for all adults 60 years and older was issued in 1998. In 2012, the federal state of Saxony issued a recommendation for sequential vaccination with PCV13 followed by PPV23 for adults over 60. The same sequential recommendation is in place for adults with underlying risk factors [24]. While uptake for the infant program is high (though schedule adherence is low) [25], uptake for adults is rather dismal: preliminary data from a vaccination status inquiry for older adults in Germany indicated that 26% of adults with IPD had been vaccinated, and a study from 2013 found similar results with 31% [26]. A particular challenge when attempting to provide adequate vaccination coverage to protect all age groups is the aging population of Germany, a graphical depiction of which is shown in **S1A Fig**.

This study describes the effects of the introduction of childhood pneumococcal conjugate vaccination on invasive pneumococcal disease among adults in Germany, and discusses the value of herd protection as opposed to direct vaccination of older adults.

## Materials and methods

### Study materials

The German National Reference Center for Streptococci (GNRCS) has conducted surveillance for IPD in Germany since 1992 (adults) and 1997 (children), using a laboratory-based approach. IPD cases were defined as *Streptococcus pneumoniae* isolates from blood, cerebrospinal fluid or any other normally-sterile body fluid. Microbiological diagnostic laboratories from all over Germany send isolates of IPD cases to the GNRCS on a voluntary basis. In total, over 400 laboratories have participated, including large, nationally-operating commercial labs. Participating laboratories are located in all German federal states, and the number of laboratories per federal state correlates to the different population densities of the states. The surveillance system has been described in detail in our previous work [27]. Briefly, the surveillance system has been improved over the years, with the final improvement, the installation of a web-based reporting tool (Pneumoweb, www.rki.de/pneumoweb) in January 2007. No structural changes have been made to the surveillance system since that time. The voluntary surveillance system was estimated to report over 50% of all IPD cases in Germany [28, 29].

### Characterization of isolates and serotyping

Species identification was performed using bile and optochin testing. In dubious cases, PCR analysis of several genes was performed (*ply*, *lytA*, *sodA*, 16S-rRNA). As a last resort, MLST was performed. Pneumococcal isolates were serotyped by Neufeld’s Quellung reaction using type and factor sera provided by the Statens Serum Institut, Copenhagen, Denmark [30]. Isolates were considered non-typeable when there was no reaction with any of the antisera, but isolates were still clearly characterized as pneumococci using all above mentioned methods.

### Statistical methods

**Fig 1:**
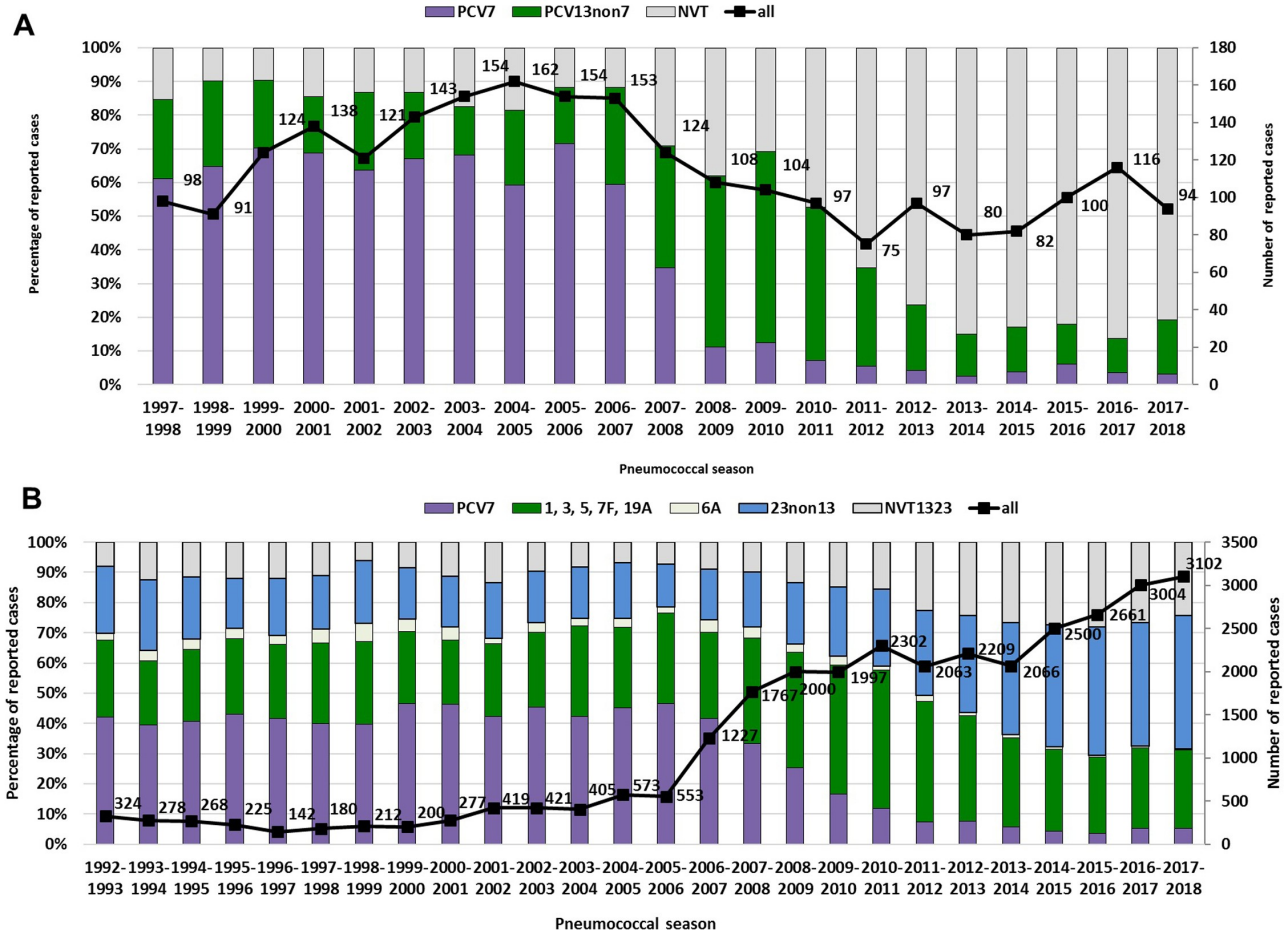
Theoretical serotype coverage of PCV7 and PCV13 among (A) children < 2 years of age and (B) PCV7, PCV13 and PPV23 among adults ≥16 years of age with IPD in Germany. NVT1323: serotypes not included in PCV13 or PPV23. Bars correspond to left vertical axis, line corresponds to right vertical axis. Stacked bar for PPV23 serotypes should be interpreted without the bar for serotype 6A.

**Fig 2:**
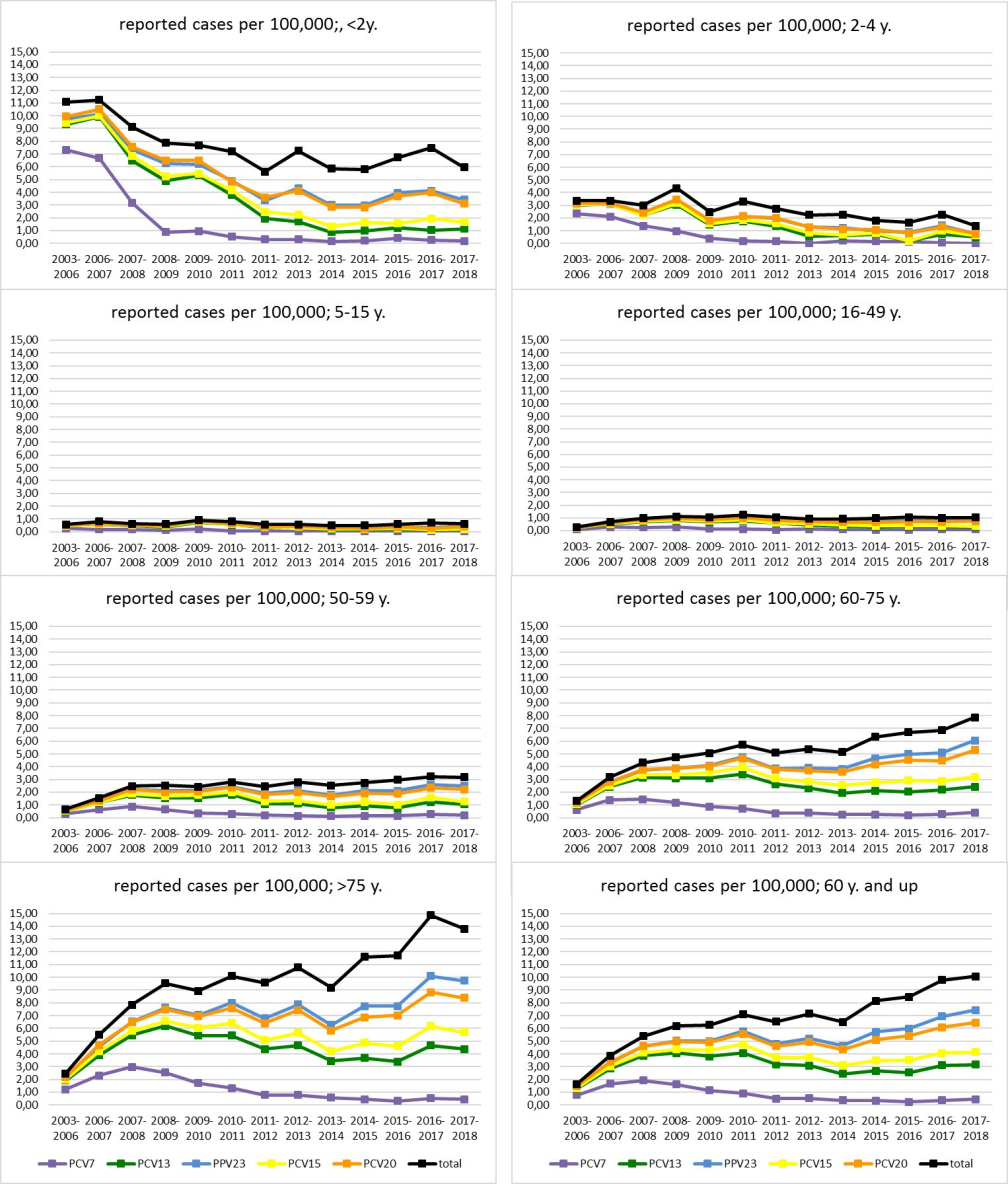
Reported cases of IPD per 100,000 population. All cases of invasive pneumococcal disease reported to the German National Reference Center for Streptococci between 1 July 2006 and 30 June 2018, per 100,000 population of Germany.

**Fig 3:**
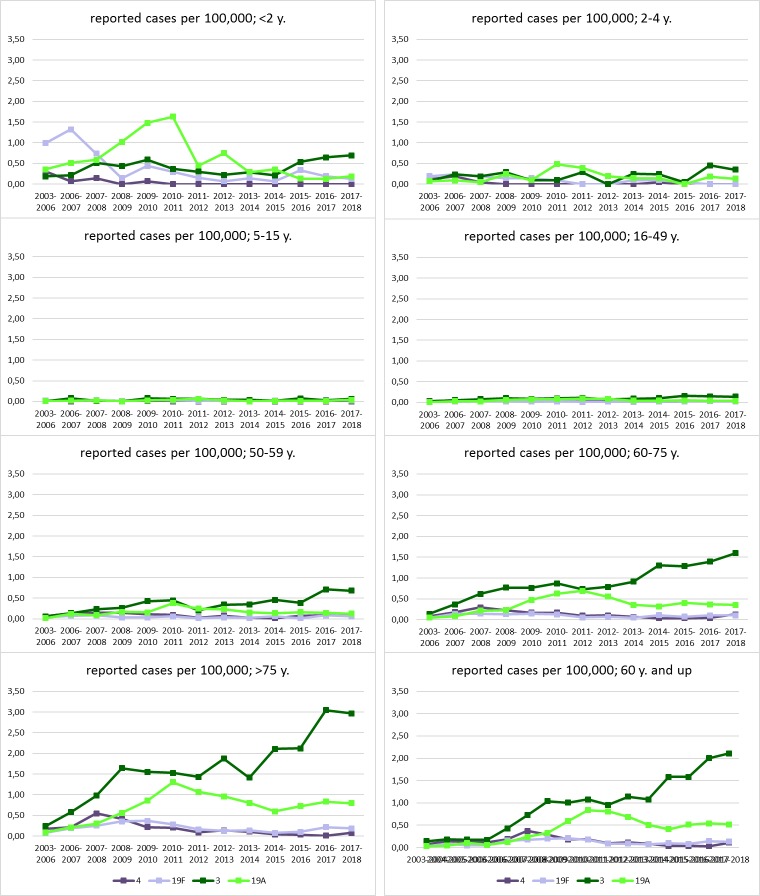
Reported cases of IPD per 100,000 population of PCV13 serotypes with a residual prevalence of >1% in 2017/18.

**Fig 4:**
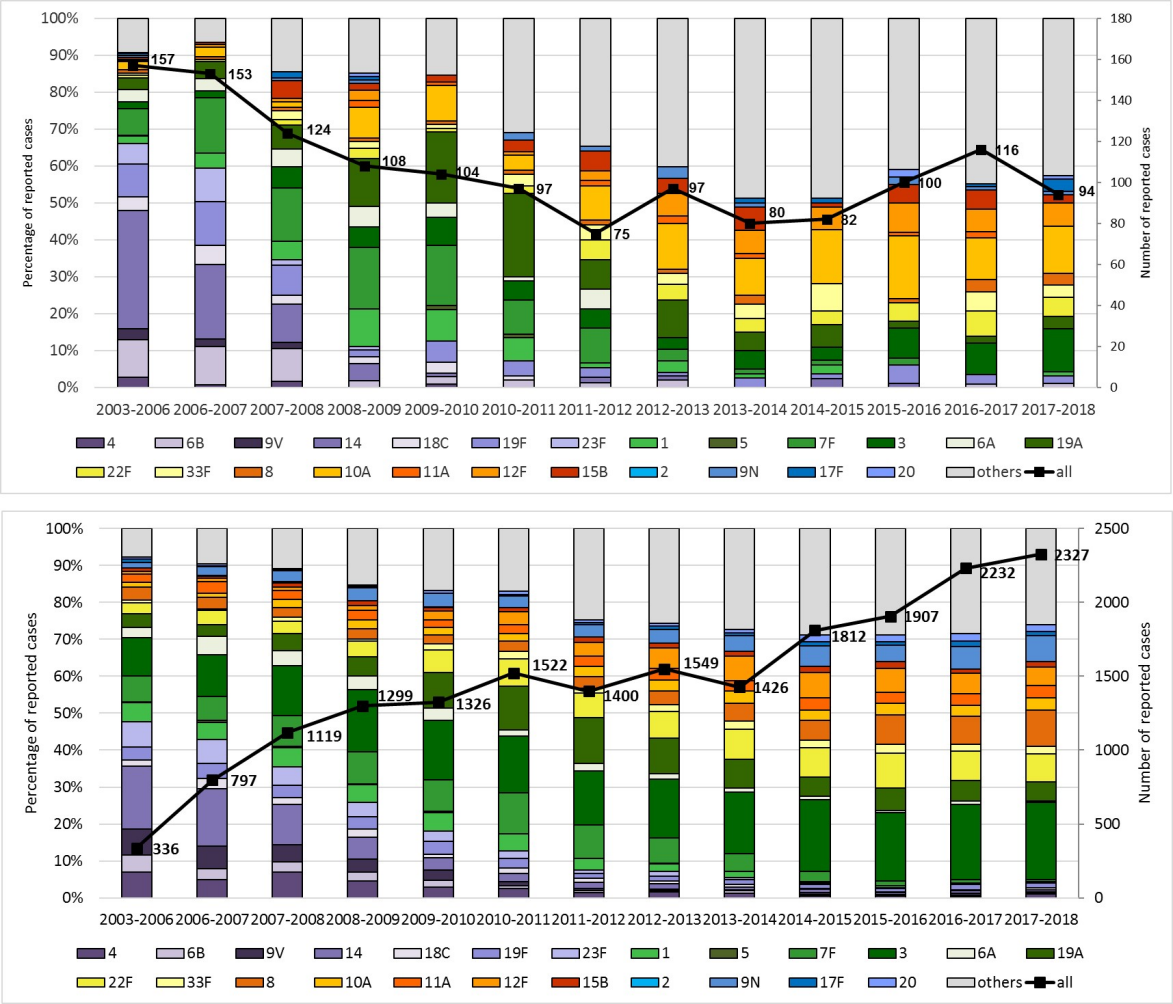
Coverage of current and future vaccine formulations among children <2 years (top) and among adults 60 years and older (bottom) with IPD in Germany. PCV7 (purple), PCV13non7 (green), PCV15non13 (yellow) and PCV20non15 (orange). PPV23 serotypes not in PCV20 (blue), Serotype 6A (white). Exact values for individual serotypes are included in **S2 Table**. Data for 2003–2006 are composite numbers of the three epidemiological years, divided by three.

Cases were grouped per epidemiological year (from July to June of consecutive years) because of known infection clusters during winter [31]. Serotype coverage information is included for the complete surveillance period, shown in **Fig 1.** The period from July 2003—June 2006 summarizes three epidemiological years in which the universal vaccination recommendation for children was not yet in place, and is included to provide a snapshot of the pre-vaccination period. A composite showing the mean values for these three epidemiological years is shown in **Figs 2, 3**and **4**(i.e. the total number of cases of the epidemiological years 2003–2004, 2004–2005 and 2005–2006, divided by three). Population data were provided by Destasis, the German Federal Statistical Office. The serotypes covered by PCV7, PCV13 and PPV23 as well as the two PCVs in development, PCV15 (PCV13 serotypes + 22F and 33F) and PCV20 (PCV13 serotypes + 22F, 33F, 15B, 12F, 11A, 10A and 8) are displayed in the figures in order to provide a preview of the coverage and potential impact of new vaccine products across various age groups in Germany [32] (https://www.clinicaltrials.gov/ct2/show/NCT03760146?term=B7471007&rank=1).

Serotype dynamics are shown as reported IPD cases per 100,000 population across several age groups: <2 years, 2–4, 5–15, 16–49, 50–59, 60–75 and >75, with an additional group of adults 60 and older, to take the German vaccination recommendation into account [24]. Five-year projections for PCV13 serotype IPD cases reported per 100,000 for adults 60 and older following PCV13 vaccination were made with the linear trendline from the population of adults 60 and older during the study period for the denominator (**S1B Fig**) and the logarithmic trendline of the reported PCV13 serotype IPD cases from the <2 age group for the numerator (**S2 Fig**), with the latter slope reduced by 20% to account for the difference between effectiveness of PCV13 in infants [25] and the effectiveness reported in adults for IPD [33]. For comparison purposes, a linear trendline for PCV13 serotype IPD in people 60 and older over the five most recent years of the study period is also shown. Proportional differences between reported cases before and after vaccination were described with Fisher’s exact test, and Poisson confidence intervals for rates were determined using the epiDisplay package in R (version 3.4.0, R Foundation for Statistical Computing, Vienna, Austria, 2017). P values less than 0.05 were considered statistically significant. Figures and tables were prepared with Microsoft Excel and Microsoft Publisher 2016.

### Ethical statement

An ethical approval was not required since the study was performed with *Streptococcus pneumoniae* isolates that resulted from routine microbiological diagnostic procedures as requested by the treating physician. No additional biological specimens were taken for the purpose of this study. Specimens were anonymized and only data on year and month of birth, sex, vaccination status, type of specimen, and hospital/laboratory where the case was diagnosed were registered.

## Results

Since 1992, 35,956 cases of IPD have been reported to the GNRCS: 4,581 from children under 16 (1997–2018; children <2: 2,415) and 31,375 from adults 16 years and older (1992–2018; adults ≥60: 21,676).

### Reduction of IPD among vaccinated children

Before the general recommendation for childhood conjugate vaccination (1997–2006), PCV7 serotypes made up between 60 and 70% (PCV13: 80–90%) of IPD cases among children < 2 years of age. After the start of vaccination in June 2006, PCV7 serotypes reduced to about 10% in only two epidemiological years’ time, and in 2017/18 made up 3.2% of IPD cases. PCV13nonPCV7 serotypes increased to up to nearly 60% of IPD cases among children < 2 years of age after PCV7 vaccination started, but decreased after the introduction of higher valent vaccines to 12.5% in 2013/14 and have remained stable (10–16%) since then. The number of reported cases has decreased from over 150 in the three years before the start of childhood vaccination (2003–2006), to around 100 in the last three seasons (2015–2018) (**Fig 1A**).

### Herd protection among adults

Among adults, coverage of PCV7 has been between 40 and 45% from 1992–2006 (PCV13: 55–60%, PPV23: 85–90%). In 2007/08, coverage started to decrease, falling to 5.8% in 2013/14, and it has remained between 3.5 and 5.2% since. PCV13nonPCV7 serotypes increased from 25–30% in 1992–2006 to 47% in 2010/11 and then decreased to 28% in 2014/15 and have remained between 25 and 27% since. PPV23 coverage has been between 80–90% since 1992, and started to decrease in 2010/11 to around 75%. Numbers of reported cases, particularly in adults, have increased over the surveillance period, largely due to enhancements of the surveillance system, but also because of the aging population. After the start of Pneumoweb in 2007, numbers of reported cases remained between 2000 and 2300 till 2014. From 2014/15 to date, case numbers increased again, reaching 3102 reported cases in 2017/18 (**Fig 1B**). In the further analyses, the epidemiological years 2003–2006 are taken as a snap-shot for the pre-PCV period for both children as well as adults.

### Serotype dynamics in reported cases per 100,000

The total and vaccine-type reported cases of IPD per 100,000 population can be found in **Fig 2**.

The reported IPD cases per 100,000 for four PCV13 serotypes with a residual prevalence of >1% in 2017/18 (4, 19F, 3, and 19A) across several age groups appear in **Fig 3**.

Total reported IPD cases per 100,000 children under 2 years of age dropped from 11.09 in the prevaccination period to 5.61 in 2011/12 (P = 6.47 x 10^−7^) and have remained steady at this level through 2017/18 (5.94, P = 0.76; pre-vaccination period versus 2017/18 P = 1.29 x 10^−6^). PCV7 serotypes have almost disappeared (0.19, from 7.34 per 100,000 prior to vaccination, P = 5.73 x 10^−30^) and PCV13 serotypes are low (1.14, from 9.32 per 100,000; P = 9.35 x 10^−26^). PCV13 coverage among children under two is currently 19%; PCV15 and PCV20 would increase coverage by 9% points (to 28% coverage) and 33% points (to 54% coverage), respectively. PCV7 serotypes have also almost completely disappeared from the two older children age groups (2–4 and 5–15), with reductions from 2.35 to 0 (P = 7.99 x 10^−17^) and 0.25 to 0.02 (P = 5.81 x 10^−5^) respectively, and PCV13 serotypes show a similar decrease (3.02 to 0.49, P = 1.64 x 10^−11^ and 0.47 to 0.17, P = 7.78 x 10^−4^).

In the adult age groups, we observed an increasing number of reported cases per 100,000 with increasing age: in adults 16–49, the pre-vaccination period had a total IPD reported cases rate of 0.28 per 100,000, rising to 1.02 (P = 3.31 x 10^−38^) in the 2017/18 epidemiological year; reported IPD cases in adults 50–59 rose from 0.66 to 3.18 per 100,000 (P = 1.64 x 10^−45^); adults 60 to 75 rose from 1.32 to 7.85 per 100,000 (P = 2.10 x 10^−169^); reported IPD cases in adults 75 and over rose from 2.45 to 13.81 per 100,000 over the study period (P = 3.16 x 10^−129^).

For adults 60 and over, the group targeted by the pneumococcal vaccination recommendation in Germany, the total reported IPD cases rose over the study period from 1.64 to 10.08 per 100,000 (P = 1.02 x 10^−315^). PCV7 serotype IPD in this age group was consistently low, declining from 0.78 to 0.43 cases per 100,000 over the study period (P = 1.97 x 10^−6^). Like in children under 2, where PCV7 type IPD has dropped to 3% of reported cases, PCV7 type IPD continues to represent slightly over 4% of the reported cases in adults over 60.

PCV13 serotype IPD in adults over 60 started at 1.26 cases per 100,000 prior to any PCV recommendation, and rose to 4.05 prior to the arrival of PCV13 (P = 3.32 x 10^−71^). After the onset of PCV13 vaccination in infants, there was an initial drop in PCV13 serotype IPD in adults 60 and over, to 2.44 cases per 100,000 in 2013/14 (P = 1.13 x 10^−20^), but since then, reported cases in this group have been steadily rising and in 2017/18 reached 3.17 per 100,000 (P = 3.92 x 10^−6^; pre-vaccination period versus 2017/18 P = 1.72 x 10^−41^), or 31% of all reported IPD cases in adults 60 and older. These vaccine-type increases have been led, far and away, by serotype 3 IPD, which by itself rose from 0.17 reported cases per 100,000 to 2.11 per 100,000 over the study period (P = 1.35 x 10^−91^), and constituted 21% of reported IPD cases in this age group.

Serotype 3 has also increased among children <2 years of age (0.19 to 0.70, P = 0.06), with many of these cases appearing in non-vaccinated (2011–2018: 21/44 (47.7%)) or incompletely vaccinated children (5/44 (11.4%); 8/44 (18.2%) occurred in children vaccinated according to age, and for 10/44 (22.7%) vaccination status could not be ascertained). In children 2–4 and 5–15 years old, reported case numbers are very low, as are case numbers in younger adults (16–49). However, reported cases per 100,000 of IPD with serotype 3 increased from 0.06 to 0.68 in adults 50–59 (P = 3.54 x 10^−16^), from 0.14 to 1.60 in adults 60–75 (P = 2.98 x 10^−48^) and from 0.24 to 2.96 in adults >75 (P = 6.42 x 10^−41^), showing a clear increasing trend with age. Over the past five years, serotype 3 has become a major contributor to the burden of disease, increasing from 6.50 reported cases per 100,000 to 10.08 over the last five years, P = 5.97 x 10^−40^.

Serotype 19A reduction among children <2 years of age has been substantial (from 1.63 per 100,000 in 2010/11 to 0.13 in 2015/16, P = 1.08 x 10^−5^), but has plateaued in the last three epidemiological years (0.13, 0.13 and 0.19). Similar to this, we see a reduction of serotype 19A cases in adults from 2011/12 to 2014/15 in all four adult age groups, but then a plateau in the last three epidemiological years. Serotypes 19F and 4 also seem to persist among adults, but at very low levels (0.01–0.02). The other PCV13 serotypes have all decreased to a prevalence of under 1%.

### Coverage of current and future vaccine formulations

The serotype coverage of different vaccine formulations for adults 60 years and older with IPD is shown in **Fig 4**. As mentioned, the coverage of PCV7 serotypes has strongly decreased over the study period. The six extra serotypes in PCV13 increased after the onset of childhood PCV7 vaccination and then decreased with the introduction of PCV13. However, serotypes 3 and 19A have persisted. When extending the analysis to the two additional serotypes in PCV15 (22F, 33F), we observe a gain in coverage over time, resulting in 10.4% extra coverage as compared to PCV13 over the last three epidemiological years. Concerning the additional serotypes in PCV20 compared to PCV15 (8, 10A, 11A, 12F, 15B) another 21.9% extra coverage could be gained. The four serotypes that are in PPV23 only (2, 9N, 17F, 20) covered around 8.9% in the last three epidemiological years. Similar values are found when considering adults 16 years and older (9.5%, 24.0%, 8.9%).

Among children under 2 years of age, when considering the last three epidemiological years, the coverage gain with PCV15 would be comparable to adults (+8.5%). However, PCV20 would add another 28.0% to that, which is higher than among adults. The extra PPV23 serotypes are less prevalent among young children (+3.7%).

#### Projections of IPD in adults

**Fig 5:**
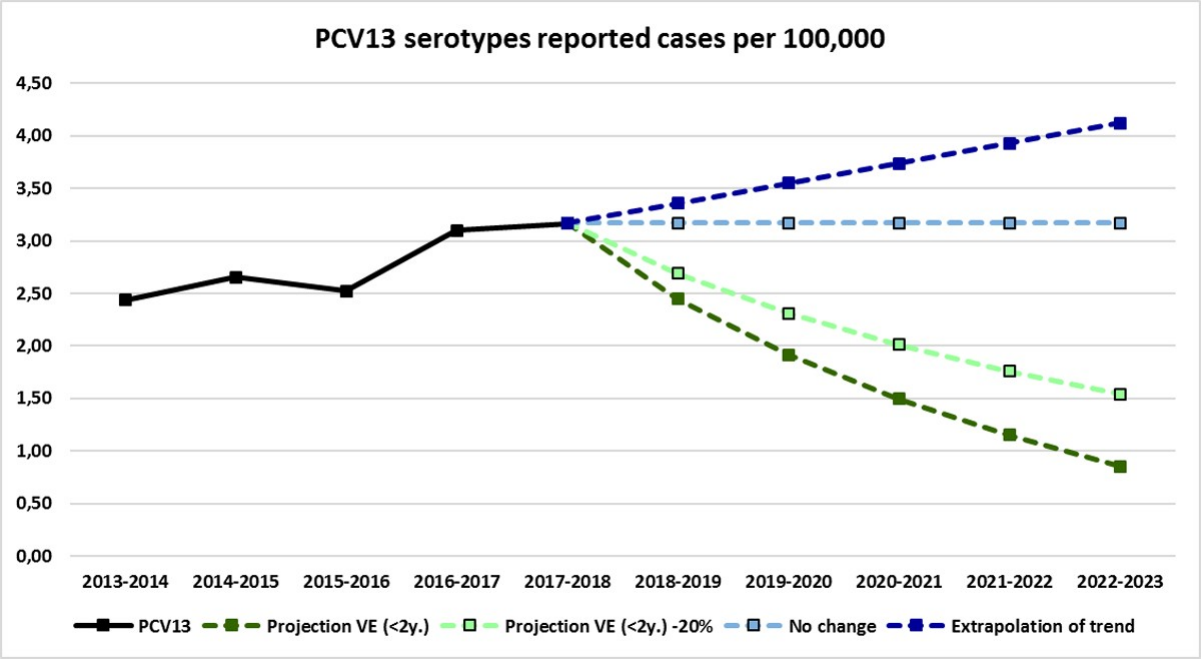
Five-year projections for cases of invasive pneumococcal disease per 100,000 population, with and without a pneumococcal conjugate vaccine program for adults. A linear trendline (y = 0.1903x+2.2067, R^2^ = 0.80) from reported cases of IPD occurring from 2013–2018 in adults over 60 was used to extrapolate reported case rates for the next five years (in dark blue). The data from **S1 Table** are shown here in light green to highlight the potential impact of direct protection on IPD in the older adult population in Germany.

Five-year projections for potential reductions in reported cases of PCV13-type IPD after onset of a high-uptake PCV13 program for adults over 60 years of age in Germany are shown in **Fig 5**.

The most recent epidemiological year, 2017/18, had 731 PCV13 serotype IPD cases (3.17 cases per 100,000), in line with an increasing trend established in 2013/14. If no changes are made in the adult vaccination recommendation, we can either assume the situation to stabilize at around 3.2 cases per 100,000 (light blue line), which could result in 767 cases of IPD in adults 60 and over for the 2022/23 epidemiological year. If, on the other hand, trends from the last five years continue, PCV13 serotype IPD in adults 60 and older could reach 997 cases per year by 2022/23 (dark blue line). With a successful PCV13 adult vaccination program, IPD incidence among adults could be reduced. Assuming the same proportional reductions in IPD cases we observed among children <2, this would result in 0.85 cases per 100,000 in 2022/23 (dark green line). Assuming a more realistic 20% lower effectiveness of PCV13 vaccination among adults, based on a 91% effectiveness found for PCV13 against vaccine type IPD in children <2 years of age in Germany [25] and a 75% efficacy for PCV13against vaccine type IPD among adults [33], IPD could still be reduced to 1.54 cases per 100,000 adults, which would fall to 372 cases in the 2022/23 surveillance year (light green line).

## Discussion

In this study, we describe a limited herd protection effect of the infant pneumococcal vaccination program on IPD among adults in Germany, with a specific focus on the age group currently targeted by the German pneumococcal (PPV23) vaccination recommendation: adults 60 and older.

The study has several limitations. As with other analyses using the GNRCS collection, the analyses described here correspond to voluntarily-reported samples sent in from diagnostic laboratories throughout Germany. Although the surveillance system has been stable for the last decade, it is of course possible that increased awareness or reporting bias could influence the sampling distribution. See **S3 and S4 Figs** for the reporting patterns from the top five contributing laboratories from 2007–2018. A capture-recapture surveillance system for IPD in children has been in place in Germany since 1997, and indicence estimates produced by this system support that, since 2007, more than half of IPD cases in Germany have been reported, which is consistent with a GNRCS audit for adult IPD cases [29, 34]. Blood culture practices in Germany have also changed over the course of the study, with approximately twice as many occurring in ICUs in 2015 as did in 2006 [35]. Obviously, a demographic shift towards an older population may also result in increased rates of IPD, and indeed, the sample distribution of the GNRCS collection has shown an increase in the proportion of samples originating from IPD cases in adults over 80 and over 90 years old over the study period, however, the population at risk for IPD is therefore more vulnerable than previous estimates may have indicated. Additionally, when describing possible future rates of reported IPD, there are several variables (carriage duration, transmission patterns, underlying illnesses, etc.) that we have not taken into account. Therefore, the data presented in **Fig 5**should only be considered a rough estimate, not a precise and all-encompassing model of potential vaccine impact. However, the figure neatly illustrates that IPD policy seems to be approaching a fork in the road: either the adult pneumococcal vaccination program will continue with uninspiring uptake and accordingly poor results, or some level of effective engagement can occur, resulting in decreased rates of invasive disease.

At the onset of the infant vaccination program, modelling studies [21] predicted a long-term protective effect, not only for the directly vaccinated children < 2 years of age, but also for non-vaccinated older children and for adults. The latter age group is of particular interest, due to an increasing burden of pneumococcal disease with increasing age. Hence, a recommendation for vaccination with pneumococcal polysaccharide vaccine (PPV23) has been in place since 1998 [36].

The infant pneumococcal vaccination program in Germany resulted in the overall decline in both vaccine-type disease and the overall reported rate of IPD in directly protected children under 2 as well as in older children [37]. Among adults, there has been a significant decline in several -PCV serotypes since the start of the infant vaccination program, however, aging of the population and the overall differences in serotype distribution between the age groups, particularly the meteoric rise of serotype 3, have resulted in an increase in the burden of pneumococcal disease among older age groups in Germany.

PCV13 serotypes represented 31% of all reported IPD in adults 60 and older in 2017/18. Hanquet *et al*. describe a residual percentage of PCV13 serotypes among adults 65 years and older of 20–29% in 6 European countries after five years of using PCV13 for their childhood immunization program [16]. A much lower percentage of residual PCV13 serotypes among adults (17%) is reported from the US [38]. And, whereas our study shows an overall increase in IPD among adults 60 years and older in Germany, Hanquet *et al*. report a 14% (CI: -4% to 30%) decline in IPD among older adults in countries using PCV13, but found an overall increase in the last year of their study (2015). In the US, the reported decrease in adult IPD was 12–32% in 2013 [15].

Among children under 2, the PCV13 serotypes have largely, but not completely, disappeared, with only serotypes 3, 19A and 19F continuing to account for 17% of IPD, though this is, for a large part, in unvaccinated and incompletely-vaccinated children. Among adults, the PCV13 serotypes persisting in more than 1% of IPD cases are 3, 4, 19A, and 19F. With the exception of serotype 4, the persistent serotypes are common between adults and children.

Reported post-PCV13 vaccination IPD incidences among adults over 65 in the US, the UK, and France ranged between 20–30 cases per 100,000 in 2015 [39, 40]. In the last year of our study, 2017–2018, we had 10 reported IPD cases per 100,000 in adults 60 years and older. Since we estimate that we receive approximately half of the IPD cases in Germany, doubling our reported cases puts us in line with the international community.

Many countries that introduced childhood vaccination programs have reported sharp decreases of PCV13 serotypes in IPD, but also residual, although very low, levels of vaccine types, often including serotypes 19A, 19F and 3 [41]. This appears to be due to a lower effectiveness of PCV13 against these serotypes. In particular for serotype 3, reported effectiveness varies between 26% and 79% [42]. In our study, over 75% of IPD cases with serotype 3 in children under 2 years of age for which the vaccination status was ascertained occurred in unvaccinated children. Among the older children (2–15 years of age), 40–45% were not vaccinated. This seems to indicate on one hand that herd protection against serotype 3 is less effective than for the other serotypes, resulting in continued transmission. Direct protection seems less effective as well, resulting in more vaccinated children getting serotype 3 IPD in the older age group.

Among adults, serotype 3 has been increasing since the start of childhood vaccination with PCV7 and the switch to PCV13 has not changed this trend. Our data seem to indicate that there is no herd protection against serotype 3, neither in older children nor in adults. There could be several reasons for this. Immunogenicity data from the licensing information of PCV13 have already shown that the vaccine induces lower antibody levels and lower opsonophagocytic titers against serotype 3 as compared to other serotypes [43]. A reason for this could lie in the characteristics of the serotype 3 polysaccharide. The serotype 3 capsular polysaccharide has only one repeating sugar unit, forming a very thick layer around the cell, making colonies appear slimy on blood agar plates. And, when in the bloodstream, serotype 3 *S*. *pneumoniae* cells are also capable of shedding saccharide subunits, thus scavenging antibodies [44]. Lower levels of antibodies probably also lead to a lower mucosal immunity, and hence a lower effect on carriage [45]. Added to that, serotype 3 shows low levels of carriage in young children [9]. And when a serotype is not carried by children, it can also not be transferred from these children to older adults. It seems therefore more likely that serotype 3 is not exclusively transferred from children, but rather circulates among older adults. And with the disappearance of other PCV13 serotypes due to effective herd protection, serotype 3 can now occupy the vacated nasopharyngeal niche. Carriage levels among adults have been reported to be low (2–5% [3]), though varying methods for detecting carriage can result in a variety of carriage rate estimates. Considering that carriage is transient, assuming a one month duration of carriage could still have a total of 24–60% of adults having carried pneumococci at some point during a year. Because our reported cases all derive from culture, rather than molecular methods, which were described by Selva et al. to better detect serotype 3, it is possible that the increases in serotype 3 reported here are actually an underestimation of changes in the burden of disease [46].

Residual cases of serotypes 19F and 19A in children under the age of two also appear mostly (84%) in unvaccinated or incompletely-vaccinated children, showing a less effective herd protection. Similarly, for these two serotypes, the percentage of un- or incompletely-vaccinated children was lower among older children (57%), indicating waning direct protection.

Among adults, we observed a reduction in serotype 19F and a rise in serotype 19A after the start of PCV7 vaccination, in parallel to the effects observed among children. After the start of PCV13 vaccination, 19A levels also decreased among adults. However, levels of both serotypes have since plateaued, resulting in considerable residual levels, especially for serotype 19A. It seems that these residual levels are a direct reflection of what is observed among children, where 19F and 19A also appear to persist in IPD, implicating a continued presence of these serotypes in carriage, as has been reported from several countries [9, 47–49]. It seems that for 19F and 19A there is an incomplete herd protection effect, caused by underpowered direct protection among vaccinated children.

Serotype 4 has become rare among children, with only three reported cases in the last 10 epidemiological years. Among adults, this serotype persists in all four age groups. The low rate of serotype 4 disease in children therefore makes transfer an unlikely cause of adult IPD, which indicates possible circulation of serotype 4 among adults.

Levels of serotypes 4 and 19F are comparable in the two older adult age groups. However, for serotypes 3 and 19A, levels clearly increase with increasing age, especially for serotype 3. This seems to indicate a particular vulnerability of older adults for these two serotypes. Considering that this study only described cases of IPD, and that pneumonia is the most common pneumococcal disease among adults, and that serotype 3 plays a major role in community acquired pneumonia as well [50], a consideration of direct adult vaccination seems wholly appropriate to prevent disease in a growing and vulnerable population.

Given that the most recent life exptectancy estimate for Germany stands at 80.8 years (78.4 for men and 83.2 for women) [51], long-term protection is essential for a vaccination program targeting adults 60 and older. Waning effectiveness over time [52], as well as complications caused by frequent repeated doses [53] might make an effective adult vaccination program based solely on PPV23 infeasible. Previous studies have described serviceable effectiveness [33, 54] of PCV13 in older adult populations, while the situation for PPV23 is more conflicted [52, 55]. The issue of vaccine selection for adult programs has been the subject of much debate in recent years in Germany [20, 56]. Compliance with vaccine recommendations in older adults in Germany is poor [26, 57], which is consistent with findings in the US, France, and the UK [52, 58, 59].

Uptake of both PPV and PCV in older adult populations has been low, though PPV uptake in Spain was positively associated with regular visits to the general practitioner and receiving the influenza vaccine [60], which might provide a useful inroad for healthcare practitioners to promote vaccination against pneumococcal disease. Another way to boost uptake might be to utilize measures designed for improving uptake in children, such as establishing alternative venues where vaccination can take place, and successfully targeting the desired age group in the promotion of vaccination events [61]. Working closely to improve communication with both general practitioners and community service providers who work with older adults could provide valuable insights on how to raise the level of vaccine uptake in the targeted age group.

The two new PCV products in development could offer a considerable increase in serotype protection, both among children and adults. A large number of the emerging serotypes that have been observed are to be included in PCV15 and PCV20. There are some interesting differences in potential coverage between children and adults: over the last three epidemiological years (2015–2018), PCV13 serotypes and serotypes not included in any vaccine formulation made up about 60% of all IPD cases, however, PCV13 serotypes represented only 17% of IPD in children but 31% in adults. The two additional PCV15 serotypes would add roughly 10% coverage to both groups, but the five extra serotypes in PCV20 as compared to PCV15 would add 6% more coverage potential to children than to adults. This difference is mainly caused by the higher rates of serotype 10A IPD among children. The four serotypes in PPV23 which are not in PCV20 are more common among adults than among children, which is mainly due to the higher prevalence of serotype 9N among adults.

Several studies model the cost effectiveness of a robust, high-uptake, adult PCV program [62–64], while the actual implementation of such a program has yet to be realized. As the population of Germany ages and the rate of reported IPD continues to increase, some efforts are underway to improve vaccine uptake in older adults [57]. As shown in **Fig 5**, the situation for older adults in Germany has great potential for improvement, but in order to maximize impact, active outreach to geriatricians, home health care aides, and other community contact points may be necessary. As we wait for the arrival of third-generation vaccines, and the potential for expanded serotype coverage, improved infrastructure, contact, and delivery pathways can be established now to ensure a speedy, highly-effective rollout and maximize the protective impact on the burden of disease in this growing population.

## Conclusions

After initial decreases in IPD in all age groups following the implementation of an infant pneumococcal conjugate vaccination program, vaccine-type disease has hit a plateau in adults, particularly in older adults. The benefits of a robust vaccination program for older adults in Germany should be further explored.

## Supporting information

S1 Fig **(A) Population distribution of Germany by age group, 1997–2017.** An overview of the percentages of each age group making up the total population of Germany, showing a shift toward an increasing percentage of older residents. The population of adults 60 years and older in this time period increased from 17.9 million in 1997 to 23.1 million in 2017. **(B) German population over 60 years of age, for the post-vaccination period 2006–2018.** Total population of all German residents older than 60 years of age, measured yearly from 31 December 2006 through 31 December 2017. A linear trendline, y = 220607x+2*10^7^, R^2^ = 0.98, was used to predict population growth for IPD case projections (Fig 5 and S1 Table).
(TIF)

S2 Fig Decrease in invasive pneumococcal disease in children under two years of age following the implementation of an infant pneumococcal conjugate vaccination program in Germany. The decrease, in reported cases per 100,000 population, of invasive pneumococcal disease caused by PCV13 serotypes following the onset of the infant vaccination program, calculated from the arrival of PCV13 to the German market (2009–2010 epidemiological year). The logarithmic trendline, y = -2.004*ln*(x)+4.8521, R^2^ = 0.88, was used for the five-year case reduction projections for older adults shown in Fig 5 and S1 Table).
(TIF)

S3 Fig Reported cases of IPD in the post-vaccination period, 2007–2018. The top five contributing microbiological laboratories (anonymized) are shown in color, the counts of IPD isolates from all remaining contributing laboratories are shown in gray.
(TIF)

S4 Fig Reported cases of IPD from the top five contributing microbiological laboratories, 2007–2018. The five largest contributing laboratories (anonymized) with samples reported each year, by sample count, are shown for the post-vaccination period.
(TIF)

S1 Table Five-year projection of the reported cases of PCV13-type IPD following an emphatic pneumococcal conjugate vaccination program in adults over 60 years of age in Germany. The effect of direct protection in children is approximated by a logarithmic trendline (see **S2 Fig**). Five-year projections for reported cases of PCV13-type IPD and 95% Poisson confidence intervals are shown using this trendline with the slope reduced by 20%, to compensate for reported differences in effectiveness of PCV13 against IPD in young children [25] versus IPD in adults [33].
(XLSX)

S2 Table Serotype distribution for children and adults in the post-PCV vaccination era. Counts of reported IPD cases by serotype for the period 2007 to 2018. Counts for 2003–2006 are composite numbers of the three epidemiological years, divided by three.
(XLSX)

S3 Table Statistically significant single serotype increases and decreases in the proportions of IPD over three time periods. The statistical significance threshold was determined by the Dunn-Sidak correction for multiple testing, P = 0.002.
(DOCX)

S4 Table Raw dataset. Serotype counts by age group and year.
(XLSX)
