# Correction: Limited indirect effects of an infant pneumococcal vaccination program in an aging population

**DOI:** 10.1371/journal.pone.0228126

**Published:** 2020-01-16

**Authors:** Mark van der Linden, Matthias Imöhl, Stephanie Perniciaro

Fig 3 is incorrect. The bottom right panel, labeled “reported cases per 100,000; 60 y. and up,” is distorted. The authors have provided a corrected figure here.

**Fig 3:**
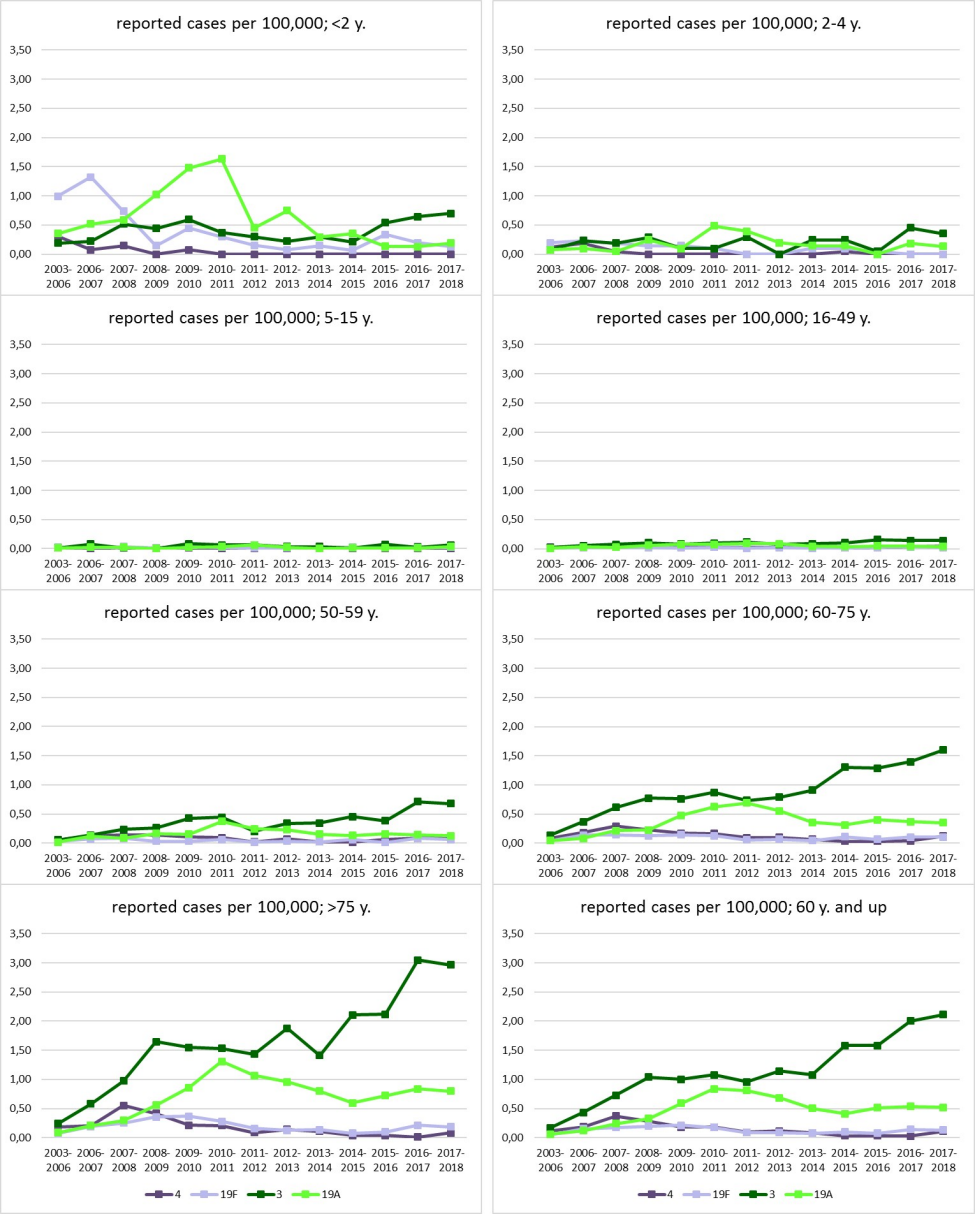
Reported cases of IPD per 100,000 population of PCV13 serotypes with a residual prevalence of >1% in 2017/18.

S4 Table is incorrect. There are data field and data row errors that occurred during final production of the file. Please view the correct S4 Table below.

## Supporting information

S4 Table Raw dataset. Serotype counts by age group and year.
(XLSX)

## References

[1] van der Linden M, Imöhl M, Perniciaro S (2019) Limited indirect effects of an infant pneumococcal vaccination program in an aging population. PLoS ONE 14(8): e0220453 10.1371/journal.pone.0220453 31369597 PMC6675109

