# Supplementary figures and images for: Limited indirect effects of an infant pneumococcal vaccination program in an aging population
Source: PLoS One. 2019 Aug 1;14(8):e0220453. doi: 10.1371/journal.pone.0220453 (PMC6675109; doi:10.1371/journal.pone.0220453)

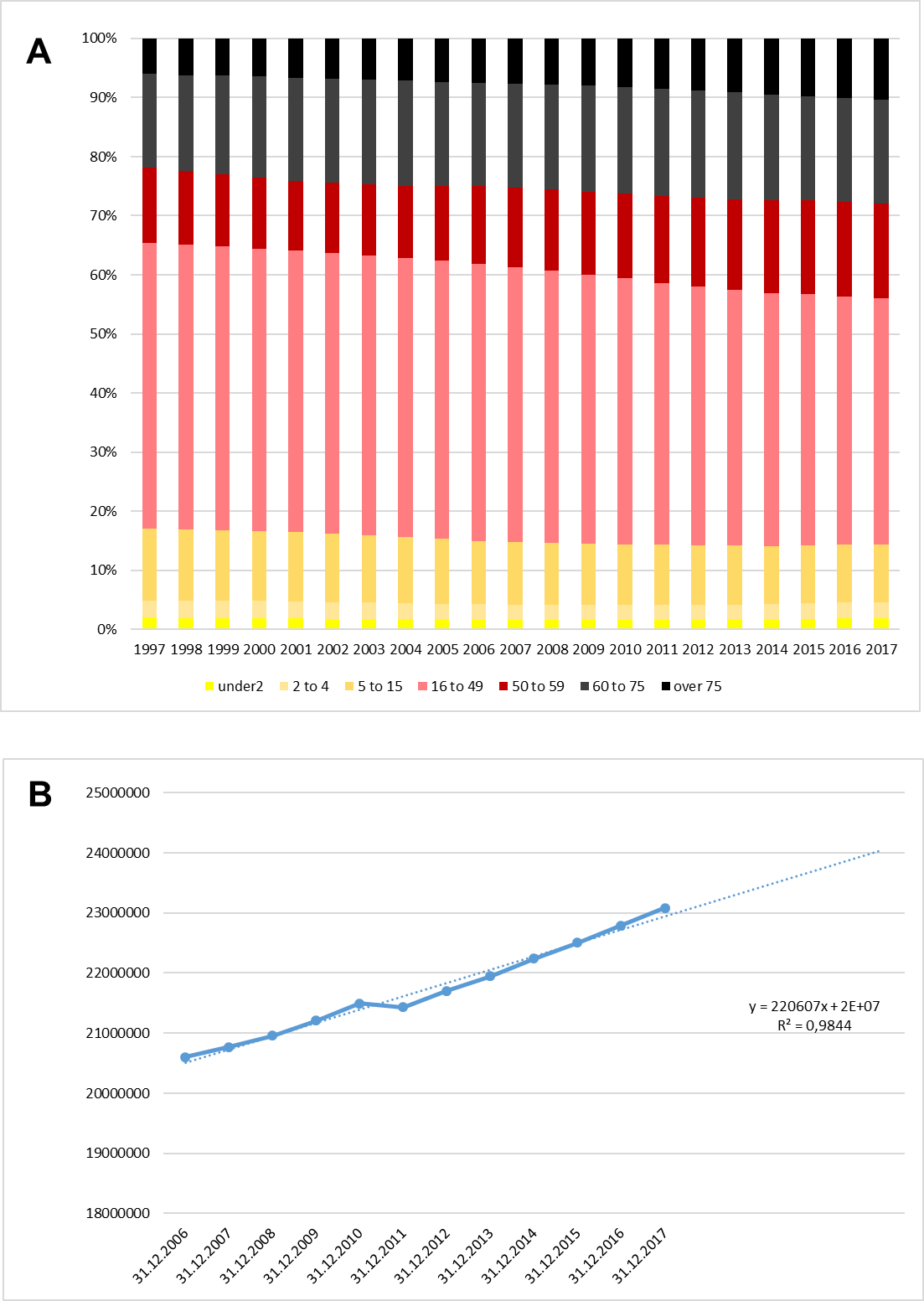

Supplement: S1 Fig — (A) Population distribution of Germany by age group, 1997–2017. An overview of the percentages of each age group making up the total population of Germany, showing a shift toward an increasing percentage of older residents. The population of adults 60 years and older in this time period increased from 17.9 million in 1997 to 23.1 million in 2017. (B) German population over 60 years of age, for the post-vaccination period 2006–2018. Total population of all German residents older than 60 years of age, measured yearly from 31 December 2006 through 31 December 2017. A linear trendline, y = 220607x+2*107, R2 = 0.98, was used to predict population growth for IPD case projections (Fig 5 and S1 Table). (TIF) [file pone.0220453.s001.tif]

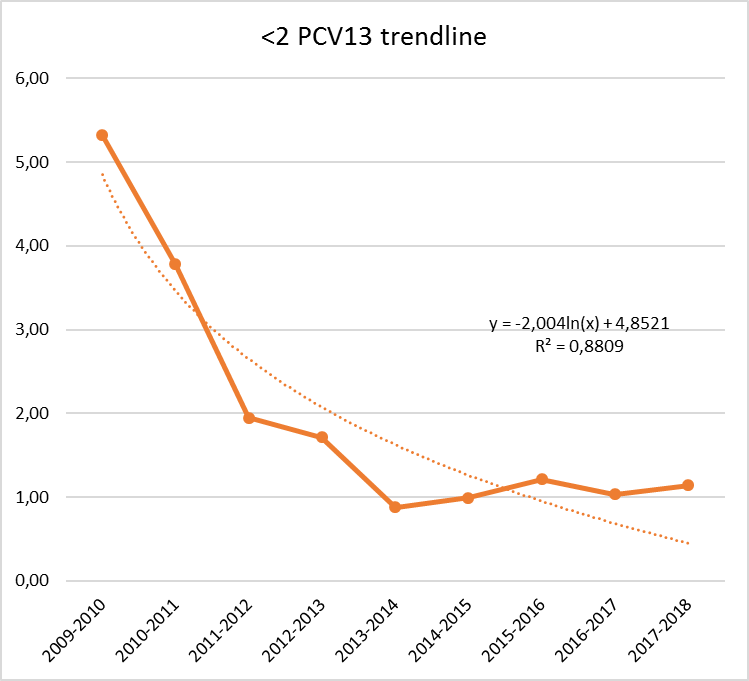

Supplement: S2 Fig — The decrease, in reported cases per 100,000 population, of invasive pneumococcal disease caused by PCV13 serotypes following the onset of the infant vaccination program, calculated from the arrival of PCV13 to the German market (2009–2010 epidemiological year). The logarithmic trendline, y = -2.004ln(x)+4.8521, R2 = 0.88, was used for the five-year case reduction projections for older adults shown in Fig 5 and S1 Table). (TIF) [file pone.0220453.s002.tif]

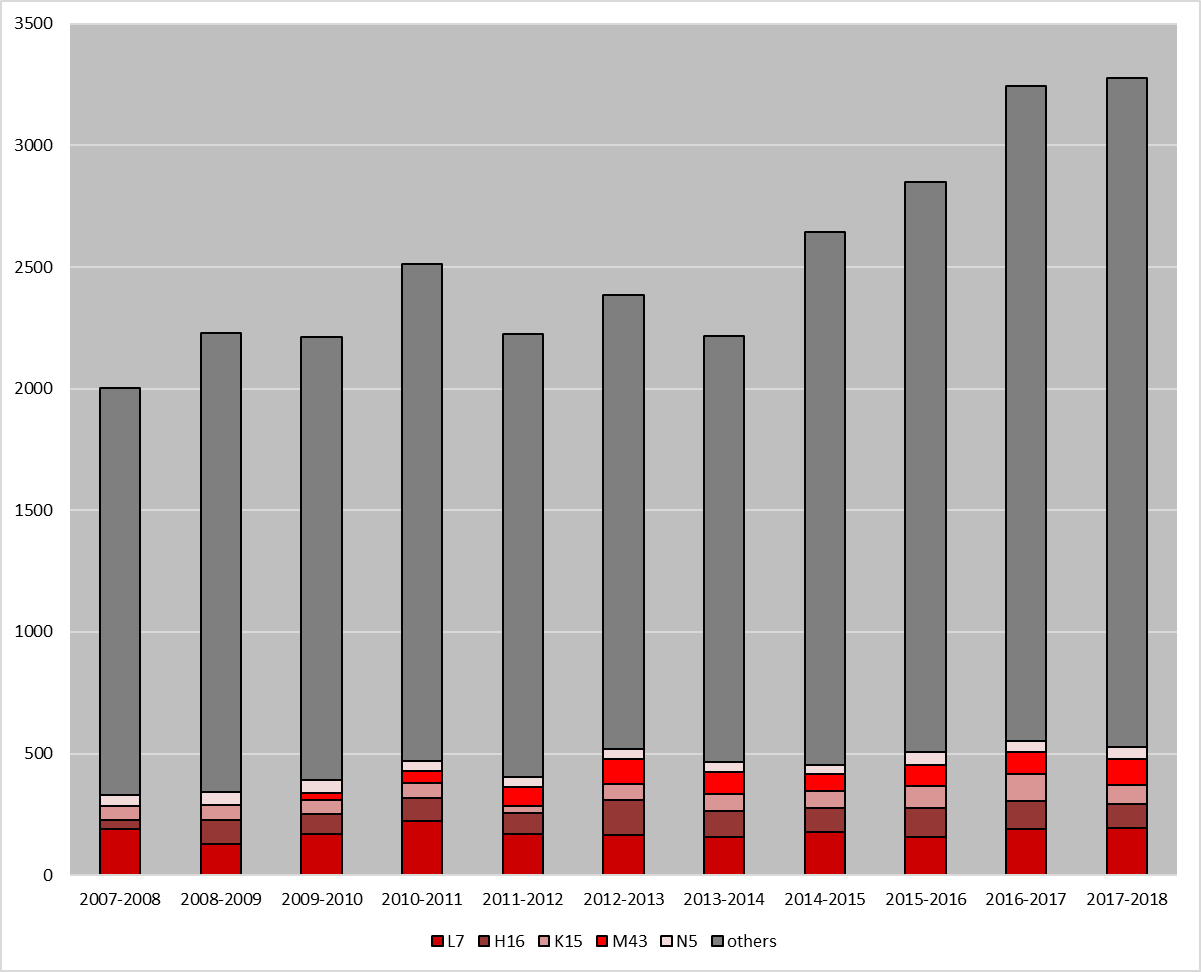

Supplement: S3 Fig — The top five contributing microbiological laboratories (anonymized) are shown in color, the counts of IPD isolates from all remaining contributing laboratories are shown in gray. (TIF) [file pone.0220453.s003.tif]

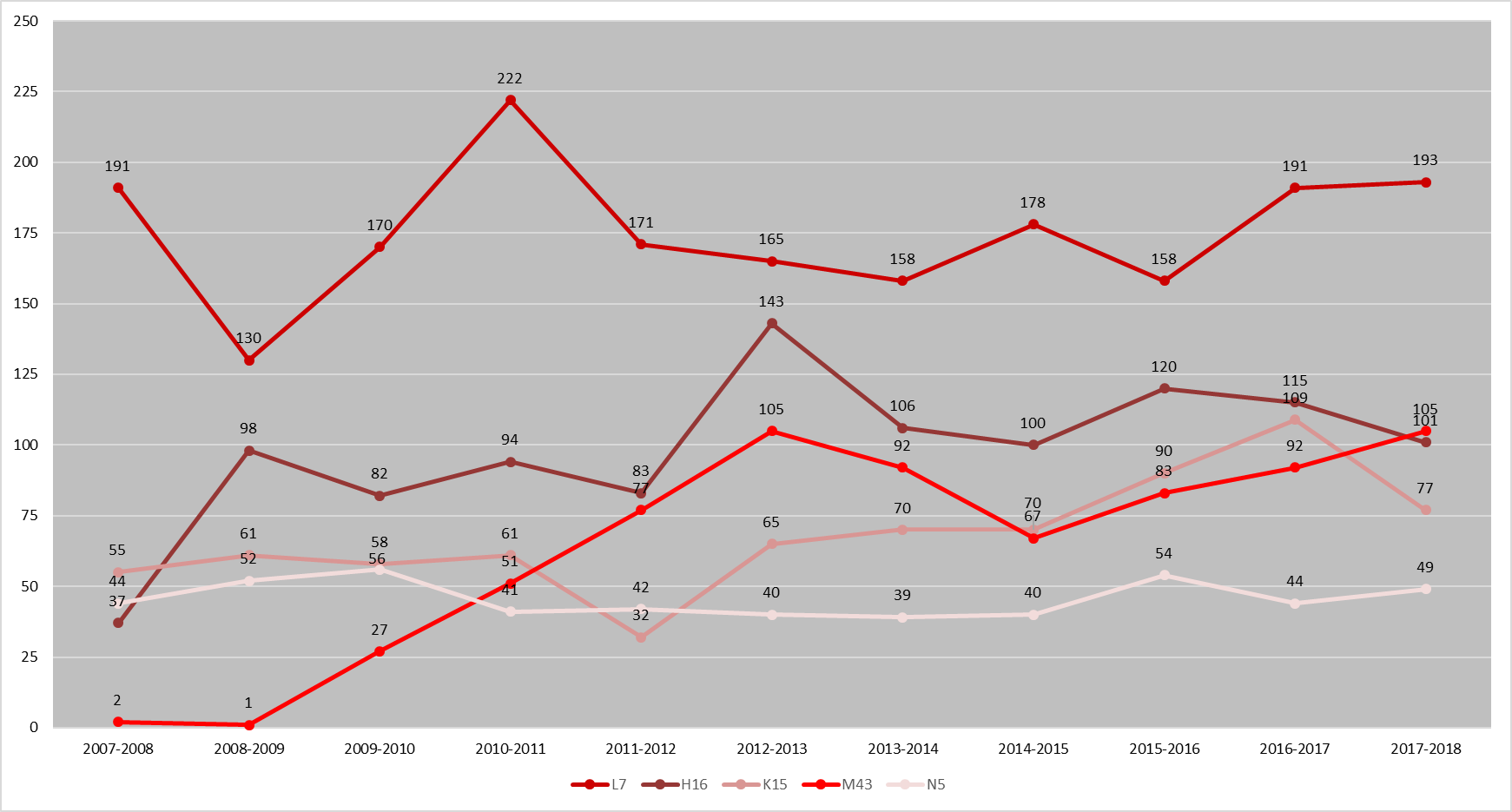

Supplement: S4 Fig — The five largest contributing laboratories (anonymized) with samples reported each year, by sample count, are shown for the post-vaccination period. (TIF) [file pone.0220453.s004.tif]
