# Supplementary material for: Limited indirect effects of an infant pneumococcal vaccination program in an aging population
Source: PLoS One. 2019 Aug 1;14(8):e0220453. doi: 10.1371/journal.pone.0220453 (PMC6675109; doi:10.1371/journal.pone.0220453)
Supplement: Supplementary file 2 [file pone.0220453.s007.docx]

Age (years)		2006-2009 vs 2009-2013	2009-2013 vs 2013-2018	2006-2013 vs 2013-2018
<2	↑	--	--	--
	↓	--	19A	19A
2-4	↑	19A	3	3
	↓	--	--	--
5-15	↑	--	--	--
	↓	--	--	--
all children	↑	19A	--	3
	↓	19F	19A	19A
16-49	↑	19A	3	3
	↓	4	19A	--
50-59	↑	19A	3	3
	↓	4	19A	4, 19A
60-75	↑	19A	3	3
	↓	4, 19F	4, 19A	4, 19F, 19A
>75	↑	19A	3	3
	↓	4, 19F	4, 19A	4, 19F, 19A
≥60	↑	19A	3	3
	↓	4, 19F	4, 19A	4, 19F, 19A
all adults	↑	19A	3	3
	↓	4, 19F	4, 19A	4, 19F, 19A
